# Providing oxygen to children in hospitals: a realist review

**DOI:** 10.2471/BLT.16.186676

**Published:** 2017-02-21

**Authors:** Hamish Graham, Shidan Tosif, Amy Gray, Shamim Qazi, Harry Campbell, David Peel, Barbara McPake, Trevor Duke

**Affiliations:** a Centre for International Child Health, University Department of Paediatrics, The Royal Children’s Hospital, 50 Flemington Road, Parkville, Victoria 3052, Australia.; b Department of Maternal, Newborn, Child and Adolescent Health, World Health Organization, Geneva, Switzerland.; c Usher Institute of Population Health Sciences and Informatics, University of Edinburgh, Edinburgh, Scotland.; d Ashdown Consultants, Hartfield, England.; e Nossal Institute for Global Health, University of Melbourne, Melbourne, Australia.

## Abstract

**Objective:**

To identify and describe interventions to improve oxygen therapy in hospitals in low-resource settings, and to determine the factors that contribute to success and failure in different contexts.

**Methods:**

Using realist review methods, we scanned the literature and contacted experts in the field to identify possible mechanistic theories of how interventions to improve oxygen therapy systems might work. Then we systematically searched online databases for evaluations of improved oxygen systems in hospitals in low- or middle-income countries. We extracted data on the effectiveness, processes and underlying theory of selected projects, and used these data to test the candidate theories and identify the features of successful projects.

**Findings:**

We included 20 improved oxygen therapy projects (45 papers) from 15 countries. These used various approaches to improving oxygen therapy, and reported clinical, quality of care and technical outcomes. Four effectiveness studies demonstrated positive clinical outcomes for childhood pneumonia, with large variation between programmes and hospitals. We identified factors that help or hinder success, and proposed a practical framework depicting the key requirements for hospitals to effectively provide oxygen therapy to children. To improve clinical outcomes, oxygen improvement programmes must achieve good access to oxygen and good use of oxygen, which should be facilitated by a broad quality improvement capacity, by a strong managerial and policy support and multidisciplinary teamwork.

**Conclusion:**

Our findings can inform practitioners and policy-makers about how to improve oxygen therapy in low-resource settings, and may be relevant for other interventions involving the introduction of health technologies.

## Résumé

**Objectif:**

Identifier et décrire les interventions qui visent à améliorer l'oxygénothérapie dans les hôpitaux des régions aux faibles ressources, et déterminer les facteurs qui contribuent à leur succès ou à leur échec dans différents contextes.

**Méthodes:**

À l'aide de méthodes d'examen réaliste, nous avons analysé la littérature et contacté des experts dans ce domaine afin d'identifier les éventuelles théories mécanistes expliquant comment pourraient fonctionner les interventions visant à améliorer les systèmes d'oxygénothérapie. Nous avons ensuite recherché de façon systématique dans des bases de données en ligne les évaluations de l'amélioration des systèmes de distribution d'oxygène dans les hôpitaux de pays à revenu faible ou intermédiaire. Nous en avons extrait des données sur l'efficacité, les processus et la théorie sous-jacente de certains projets, et avons utilisé ces données pour tester les théories candidates et identifier les caractéristiques des projets qui avaient abouti.

**Résultats:**

Nous avons examiné 20 projets d'amélioration de l'oxygénothérapie (45 articles), menés dans 15 pays. Ces projets appliquaient différentes approches pour améliorer l'oxygénothérapie et présentaient des résultats en matière clinique, technique et de qualité des soins. Quatre études de l'efficacité montraient des résultats cliniques positifs pour la pneumonie infantile, avec de grandes variations selon les programmes et les hôpitaux. Nous avons identifié les facteurs qui contribuent au succès des interventions ou qui l'entravent et avons proposé un cadre pratique décrivant les conditions essentielles que doivent remplir les hôpitaux pour administrer efficacement des oxygénothérapies aux enfants. Pour de meilleurs résultats cliniques, les programmes d'amélioration de la distribution d'oxygène doivent assurer un bon accès à l'oxygène et une bonne utilisation de celui-ci, choses qui devraient être facilitées par d'importants moyens d'amélioration de la qualité, des politiques et un encadrement solides ainsi qu'une équipe de travail multidisciplinaire.

**Conclusion:**

Nos conclusions pourront orienter les praticiens et les responsables politiques en vue d'améliorer l'oxygénothérapie dans les régions aux faibles ressources, et pourront être utiles pour d'autres interventions nécessitant l'introduction de technologies de santé.

## Resumen

**Objetivo:**

Identificar y describir las intervenciones para mejorar la oxigenoterapia en hospitales de entornos con pocos recursos y determinar los factores que contribuyen al éxito y al fracaso en diversos contextos.

**Métodos:**

Utilizando métodos de revisión realistas, se analizaron los documentos y se contactó con expertos en el campo para identificar posibles teorías mecanicistas sobre cómo pueden funcionar las intervenciones a la hora de mejorar los sistemas de la oxigenoterapia. Posteriormente, y de forma sistemática, se realizaron búsquedas en bases de datos en línea con el objetivo de encontrar evaluaciones de sistemas con oxígeno mejorados en hospitales de países con ingresos bajos o medios. Se extrajo información sobre la eficacia, los procesos y la teoría subyacente de los proyectos seleccionados y se utilizó dicha información para probar las posibles teorías e identificar las características de los proyectos que tuvieron éxito.

**Resultados:**

Se incluyeron 20 proyectos de oxigenoterapia mejorados (45 documentos) de 15 países. Estos proyectos utilizaban distintos enfoques para mejorar la oxigenoterapia e informaban de resultados clínicos, técnicos y de la calidad de la atención. Cuatro estudios de eficacia demostraron resultados clínicos positivos para la neumonía infantil, con una gran diferencia entre programas y hospitales. Se identificaron factores que contribuyen u obstaculizan el éxito, y se propuso un marco práctico que representaba los requisitos fundamentales para que los hospitales proporcionen una oxigenoterapia de forma eficaz a los niños. Para mejorar los resultados clínicos, los programas de mejora del oxígeno deben lograr un buen acceso al oxígeno y un buen uso del mismo, lo que debería verse facilitado por una amplia capacidad de mejora de la calidad, un apoyo de gestión y político sólido y trabajo en equipo multidisciplinar.

**Conclusión:**

Los resultados pueden informar a los médicos y responsables políticos sobre cómo mejorar la oxigenoterapia en entornos con pocos recursos, y pueden ser relevantes para otras intervenciones que impliquen la introducción de tecnologías sanitarias.

## ملخص

**الغرض:**

تحديد ووصف التدخلات اللازمة لتحسين العلاج بالأكسجين في المستشفيات التي تعاني من شح الموارد، وتحديد العوامل التي تسهم في نجاحها أو فشلها في سياقات مختلفة.

**الطريقة:**

لقد استخدمنا طرق استعراض واقعية لتصفح المؤلفات والتواصل مع خبراء متخصصين في المجال لتحديد آليات التدخل المحتملة لتحسين نظم العلاج بالأكسجين. عقب ذلك قمنا بالبحث وبشكل منهجي في قواعد البيانات الموجودة على شبكة الانترنت عن تقييمات متعلقة بالنظم المطوّرة للعلاج بالأكسجين في المستشفيات الواقعة في البلدان منخفضة الدخل أو البلدان متوسطة الدخل. واستخلصنا بيانات حول مدى فاعلية المشروعات المختارة وطرق معالجتها والآلية الأساسية المتعلقة بها، وقد قمنا باستخدام هذه البيانات لاختبار الآليات المرشحة والوقوف على خصائص المشاريع الناجحة.

**النتائج:**

قمنا بإدراج 20 مشروعًا لاستخدام العلاج المحسّن بالأكسجين (45 **  **دراسة بحثية) مستمدًا من 15 **  **دولة. وقد عمدت هذه المشاريع إلى استخدام أساليب مختلفة في تحسين العلاج بالأكسجين، كما قدمت المحصلات المتعلقة بالنواحي السريرية والجودة النوعية للرعاية المقدمة وكذلك المحصلات التقنية. وقد أشارت أربع دراسات لقياس الفاعلية إلى تحقيق محصلات إيجابية لعلاج الأطفال المصابين بمرض الالتهاب الرئوي، مع وجود تباين كبير بين البرامج والمستشفيات. وقد قمنا بتحديد العوامل التي قد تدعم النجاح أو قد تعمل على إعاقته وكذلك عملنا على عرض إطار عملي لوصف المتطلبات الرئيسية للمستشفيات وذلك للعمل بفاعلية على تقديم العلاج بالأكسجين للأطفال. ولتحسين النتائج السريرية، فيجب أن تعمل برامج تحسين العلاج بالأكسجين على تيسير سبل الحصول على الأكسجين وكذلك على تحقيق الاستخدام الأمثل له، الأمر الذي يمكن تسهيله بتقديم قدرة أوسع على تحسين الجودة وباستخدام إدارة قوية، وعبر توفير الدعم المقدم عبر السياسات، وكذلك من خلال العمل الجماعي بين التخصصات المختلفة.

**الاستنتاج:**

توضح نتائجنا للعاملين وصناع القرار طرق تحسين العلاج بالأكسجين في الأماكن التي تعاني من شح الموارد، وقد تكون ذات صلة وثيقة بغيرها من عمليات التدخل المتعلقة بطرح التقنيات الصحية.

## 摘要

**目的:**

旨在找出并描述改善资源匮乏地区医院氧气治疗的干预措施，并确定导致不同情况下成功和失败的因素。

**方法:**

通过务实评审方法，我们浏览了相应文献资料并联系了该领域的专家，从而明确了有关改善氧气治疗系统的干预措施如何运作的潜在力学理论。 随后，我们在网上数据库中系统地检索了中低收入国家医院改善后氧气系统的评估数据。 我们提取了有关所选项目的成效、过程及基本理论的数据，并使用这些数据对候选理论进行测试并明确成功项目的特征。

**结果:**

我们选取了来自 15 个国家的 20 个改善后氧气治疗项目（45 篇文章）。 这些项目采用了多种改善氧气治疗的方法，并报告了在临床、护理质量及技术方面的结果。 四份成效研究，通过项目与医院间的巨大差异，证明了在儿童肺炎取得了正面临床效果。 我们明确了帮助或阻碍成功的因素，并提出了一个描述对医院有效地向儿童提供氧气治疗的关键要求的可行框架。 为改善临床结果，氧气改善项目必须保证合理地获得并使用氧气，而十足的质量改善能力，强劲的管理和政策支持以及多学科协作会促进这一目标的实现。

**结论:**

我们的结果将告知从业人员和决策制定者们如何改善资源匮乏地区的氧气治疗，并可能会与涉及引进卫生技术的其他干预措施有关。

## Резюме

**Цель:**

Определить и описать мероприятия по улучшению кислородной терапии в больницах в условиях ограниченности ресурсов, а также определить факторы, которые в различных ситуациях способствуют успеху и неудаче.

**Методы:**

Используя методы прагматического обзора, авторы внимательно изучили литературу и связались с экспертами в этой области для выявления возможных механистических теорий о том, как могут работать мероприятия по усовершенствованию систем кислородной терапии. Затем был проведен систематический поиск в онлайновых базах данных на предмет оценки усовершенствованных систем подачи кислорода в больницах в странах с низким и средним уровнем дохода. Авторы извлекли данные об эффективности, процессах и положенной в основу теории выбранных проектов и использовали эти данные для проверки теорий-кандидатов и определения особенностей успешных проектов.

**Результаты:**

Авторы включили 20 проектов по усовершенствованию кислородной терапии (45 документов) из 15 стран. В них были использованы различные подходы к улучшению кислородной терапии, а также были указаны клинические и технические результаты, а также результаты относительно качества оказания медицинской помощи. В четырех исследованиях эффективности были выявлены положительные клинические результаты в случае детской пневмонии на фоне большой вариативности между программами и больницами. Авторы определили факторы, которые способствуют или препятствуют успеху, и предложили практическую основу, которая описывает основные требования для больниц с целью эффективного проведения кислородной терапии для детей. Для улучшения клинических результатов программы усовершенствования кислородной терапии должны обеспечить высокую доступность кислорода и надлежащее использование кислорода, чему должен способствовать широкий потенциал для повышения качества, а также сильная управленческая и политическая поддержка и межотраслевая совместная работа.

**Вывод:**

Практикующие специалисты и высшее руководство могут использовать полученные результаты, чтобы понять, как улучшить кислородную терапию в условиях ограниченности ресурсов. Эти результаты также могут иметь значение для других мероприятий, связанных с внедрением медицинских технологий.

## Introduction

Oxygen is an essential medical therapy that has been saving lives for over 100 years.^1^ Oxygen therapy is used not only for pneumonia and other primary lung diseases but also many other conditions that result in hypoxaemia, such as sepsis, severe malaria, status epilepticus, trauma, obstetric and neonatal conditions (respiratory distress, apnoea, asphyxia, sepsis), surgical care and anaesthesia. A systematic review estimated that, globally, hypoxaemia affects about 13% of children admitted to hospital with pneumonia, about 20% of sick neonates and 10–15% of children admitted with conditions such as malaria, meningitis or convulsions.^2^ Given that hypoxaemia is a major risk factor for death,^2^^,^^3^ oxygen therapy is important for improving child health outcomes.

Effective oxygen therapy requires prompt and accurate detection of hypoxaemia and appropriate administration of oxygen, combined with good clinical evaluation and management of the underlying condition.^4^ Improvements in the technology and affordability of pulse oximetry ‒ the standard method for detecting hypoxaemia ‒ are enhancing its accessibility for hospitals in low-resource settings.^1^ Oxygen may be supplied by oxygen cylinders, oxygen concentrators or larger oxygen plants, each of which have unique advantages and disadvantages, particularly when used in hot, humid or dusty environments.^1^^,^^5^ The World Health Organization (WHO) has produced guidelines on the clinical use of oxygen^6^^–^^8^ and oxygen equipment.^9^

Despite these advances, the availability of pulse oximetry and oxygen supplies remains limited in regions of the world where they are most needed.^10^ Furthermore, workforce limitations and health-system failures limit the ability to maintain, sustain or effectively use oxygen even when it is available.^1^ A solution to this must be multifaceted, and is likely to be context-specific.

We aimed to identify and describe interventions to improve oxygen therapy in hospitals in low-resource settings, and to determine the factors that contribute to the success or failure of interventions in different contexts.

## Methods

We used a realist review approach^11^ to study not only whether oxygen therapy interventions work, but also how and why complex programmes work in particular contexts and settings. Realist review is a theory-driven systematic review method that involves identifying key mechanistic theories about how projects might work, searching the evidence about project implementation and impact (including variability between contexts), and then testing the evidence with respect to the theories.^11^^–^^14^ Our review was prospectively registered on the PROSPERO register of systematic reviews (CRD42015032405).

### Identification of theories

We made a preliminary scan of the literature to identify potential theories to explain how improved oxygen therapy systems could impact on clinical outcomes. This resulted in a list of candidate theories, each describing a mechanism through which the intervention influences particular outcomes in particular contexts. We consulted key experts and stakeholders, including interviewing the authors of five large-scale oxygen therapy projects. Interviews were recorded and transcribed for accuracy, but were not formally analysed and were used only to assist in identifying emerging themes.

### Search strategy

We made a systematic search of online databases (MEDLINE®, Embase®, CINAHL, AIM, LILACS, the Index Medicus for the Eastern Mediterranean Region, the Index Medicus for South-East Asia Region, the Western Pacific Region Index Medicus, CAB Global Health, Health Systems Evidence, PubMed® (for e-publications) and Google Scholar (first 500 citations)) on 10 August 2016. We searched variations of keywords “child”, “oxygen concentrator”, “oxygen cylinder”, “oxygen therapy”, “oxygen delivery”, “oxygen administration” and “developing country” (MEDLINE® search; Box 1). We also searched websites (e.g. WHO and the International Union Against Tuberculosis and Lung Disease), contacted oxygen therapy experts and reviewed the reference lists of included studies to identify additional published and unpublished studies.

Box 1 **MEDLINE® search for publications on interventions to improve oxygen therapy systems in low-resource settings**

**Search run on 10 August 2016**

1. (newborn* or neonat* or infan* or preschooler* or pre-schooler* or toddler* or child* or adolescen* or pediatric* or paediatric*).af.

2. (sd or ec or is or mt or th).fs.

3. developing countries/

4. (austere or (low adj2 resource*) or (limited adj2 resource*) or transitioning econom* or emerging countr* or developing countr* or (("low income" or "middle income" or "low to middle income") and countr*) or "third world" or (underdeveloped adj countr*) or (under adj developed adj countr*) or LMIC).mp.

5. exp africa/

6. americas/ or exp caribbean region/ or exp central america/ or latin america/ or mexico/ or exp south america/

7. europe/ or exp europe, eastern/ or exp transcaucasia/

8. antarctic regions/ or exp atlantic islands/ or exp indian ocean islands/ or exp pacific islands/

9. New Guinea/

10. asia/ or exp asia, central/ or asia, southeastern/ or borneo/ or cambodia/ or east timor/ or indonesia/ or laos/ or malaysia/ or mekong valley/ or myanmar/ or philippines/ or thailand/ or vietnam/ or asia, western/ or bangladesh/ or bhutan/ or india/ or middle east/ or afghanistan/ or iran/ or iraq/ or jordan/ or lebanon/ or oman/ or saudi arabia/ or syria/ or turkey/ or yemen/ or nepal/ or pakistan/ or sri lanka/ or far east/ or china/ or tibet/ or exp korea/ or mongolia/

11. (Afghanistan or Albania or Algeria or Angola or Antigua or Argentina or Armenia or Azerbaijan or Bangladesh or Barbados or Barbuda or Belarus or Belize or Benin or Bhutan or Bolivia or Bosnia or Botswana or Brazil or Bulgaria or "Burkina Faso" or Burma or Burundi or Cambodia or Cameroon or "Cape Verde" or "Cabo Verde" or "Central African Republic" or Chad or Chile or China or Colombia or Comoros or Congo or (Cook adj Island*) or "Costa Rica" or "Cote D'ivoire" or Croatia or Cuba or "Czech Republic" or Czechoslovakia or Djibouti or Dominica or Dominican or "East Timor" or Ecuador or Egypt or "El Salvador" or "Equatorial Guinea" or Eritrea or Estonia or Ethiopia or Fiji or Futuna or Gabon or Gambia or Gaza or Georgia or Ghana or Grenada or Guatemala or Guinea or "Guinea Bissau" or Guyana or Haiti or Herzeg* or Honduras or Hungary or India or Indonesia or Iran or Iraq or "Ivory Coast" or Jamaica or Jordan or Kazakhstan or Kenya or Kiribati or Korea or Kosovo or "Kyrgyz Republic" or Kyrgyzstan or Laos or (Lao adj People* adj Democratic adj Republic) or "Lao PDR" or Latvia or Lebanon or Lesotho or Liberia or Libya or Lithuania or Macedonia or Madagascar or Malawi or Malaysia or Maldives or Mali or (Marshall adj Island*) or Mauritania or Mauritius or Mexico or Micronesia or Moldova or Mongolia or Montserrat or Montenegro or Morocco or Mozambique or Myanmar or Namibia or Nauru or Nepal or "New Guinea" or Nicaragua or Niue or Niger or Nigeria or Oman or Pakistan or Palau or Panama or "Papua New Guinea" or Paraguay or Peru or Philippines or Poland or Yemen or Romania or Russia or Rwanda or "Saint Kitts Nevis" or "St Kitts Nevis" or "Saint Vincent Grenadines" or Samoa or "St Vincent Grenadines" or "Saint Lucia" or "St Lucia" or "Saint Helena" or "St Helena" or "Sao Tome Principe" or "Saudi Arabia" or Senegal or Serbia or Seychelles or "Sierra Leone" or Slovak or "South Africa" or Solomon Island* or Somalia or "Sri Lanka" or Sudan or Suriname or Swaziland or Syria or Tajikistan or Tanzania or Thailand or Tibet or "Timor-Leste" or Togo or Tokelau or Tonga or Trinidad or Tobago or Tunisia or Turkey or Turkmenistan or Tuvalu or Uganda or Ukraine or Uruguay or Uzbekistan or Vanuatu or Venezuela or Vietnam or "Wallis Futuna" or "West Bank" or Yemen or Zambia or Zimbabwe or Zaire).mp.

12. (africa or americas or caribbean or "central america" or "latin america" or "south america" or "eastern europe" or Transcaucasia or antarctic or (atlantic adj island*) or (indian adj ocean adj island*) or (pacific adj island*) or polynesia or "central asia" or (southeast* adj asia) or (south adj east* adj asia) or borneo or mekong or "western asia" or "middle east" or "far east").mp.

13. 3 or 4 or 5 or 6 or 7 or 8 or 9 or 10 or 11 or 12

14. (oxygen adj2 (concentrator* or cylinder* or therap* or delivery or administ*)).tw,kw,hw.

15. 2 and 13 and 14

We included any evaluation of an improved oxygen therapy system involving a hospital in a low- or middle-income country (World Bank definitions at the time of the study). An improved oxygen therapy system included introduction of an improved oxygen source (oxygen concentrator, cylinder or plant) with or without: other equipment (e.g. pulse oximeters, oxygen delivery devices); education (e.g. training materials or visits); health-system or quality improvement activities (e.g. financing, supply chain, supervision). Two investigators independently reviewed the titles, abstract and full-text of the studies for inclusion, with the adjudication of a third reviewer where consensus could not be reached.

We assessed the quality of each study on two criteria: relevance (i.e. addresses the candidate theories about how improving oxygen systems lead to improved clinical outcomes); and rigour (i.e. provides credible data to reach a conclusion). Most studies contributed to the testing of a particular theory more than others. We only reported results where there were credible data.

To assess the quality of studies reporting clinical outcomes we used the Effective Public Health Practice Project quality assessment tool for quantitative studies.^15^^,^^16^

### Data extraction and synthesis

We used a data collection form adapted from the Cochrane Effective Practice and Organisation of Care (EPOC) group^17^ and process evaluation^18^ tools. For each project we extracted data on the theory (implicit or explicit); context (e.g. facility characteristics, oxygen capacity and needs); interventions (using Cochrane EPOC categories); processes (e.g. quality, fidelity to the project plan, how well it reached the intended beneficiaries); and outcomes (clinical, quality of care, cost, equipment function).

The data were recorded in a series of tables, enabling possible mechanistic theories to be explored within individual projects and between projects. After repeatedly exploring the partially developed theories, we aggregated them into major theoretical themes. We then analysed the available data to support, negate or refine the theories, thereby identifying key factors affecting the success of projects.

## Results

**Fig. 1:**
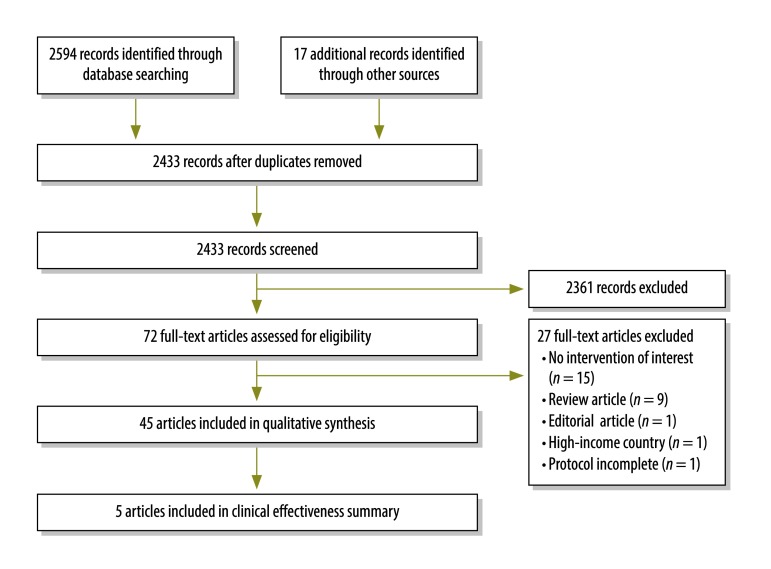
Flowchart of systematic literature search for interventions to improve oxygen therapy systems in low-resource settings

Of 2433 records screened, 72 full-text articles were assessed for eligibility (Fig. 1). We included 45 papers describing 20 projects involving an intervention(s) to improve oxygen therapy systems, from 15 countries. Data from two of the projects have not been published yet (Gray et al., Centre for International Child Health (CICH), Parkville, Australia, unpublished data, 26 January 2017 and Morpeth M et al., CICH, Parkville, Australia, unpublished data, 26 January 2017) and will hereafter be referred to as Gray et al. and Morpeth et al. Most projects (15) used non-comparative evaluation methods, one project used a contemporaneous control and four used a historical control.

The earliest projects (in the 1980s) introduced oxygen concentrators primarily for anaesthetic purposes, and evaluated their technical function and cost implications (Table 1). Since the late 1980s, seven large-scale paediatric oxygen projects have been evaluated, involving complex interventions targeting technicians, clinicians and often administrators and policy-makers (Table 2). Recently, four projects have explored the utility of improved power supplies for oxygen concentrators, including solar power systems (Table 1 and Table 2). Four large-scale effectiveness studies demonstrated reduced in-hospital mortality from pneumonia and other hypoxaemic conditions among children aged < 5 years following an intervention to improve oxygen therapy (Table 3). Due to heterogeneity in reported outcomes and insufficient comparable data, a meta-analysis was not possible.

**Table 1:** Small-scale interventions to improve oxygen therapy systems in low-resource settings, identified by a systematic literature search

Year	Country	Context	Intervention(s)	Data source(s)
1982	Democratic Republic of the Congo	Setting: single, remote mission hospital. Challenges: limited oxygen cylinder supply (logistic and cost barriers). Assets: reliable hydroelectric power supply. Aims: to achieve more reliable and affordable oxygen supply for anaesthetic use.	Equipment: one oxygen concentrator (Mountain Medical). Clinical training: not described.	A brief report provided limited data on equipment function and its various clinical uses.^19^
1985	Nepal	Setting: single military hospital in Kathmandu. Challenges: limited oxygen cylinder supply (logistic and cost barriers). Aims: to achieve more reliable and affordable oxygen supply for anaesthetic use.	Equipment: one oxygen concentrator (unspecified). Clinical training: not described.	A brief report provided data on equipment function and cost.^20^
1986	Barbados	Setting: single referral hospital. Challenges: limited oxygen cylinder supply (logistic and cost barriers); low technician capacity; no routine maintenance. Aims: to achieve more reliable and affordable oxygen supply for general use.	Equipment: one large oxygen concentrator (Rimer-Alco, 13 L/min) connected to a compressor and fed into a piped oxygen supply system. Clinical training: not described.	A historical review of anaesthesia services provided data on equipment function, cost and oxygen demand.^21^
1986	Ghana	Setting: single referral hospital (Korle Bu hospital). Challenges: frequently interrupted oxygen cylinder supply (logistic and cost barriers). Aims: to achieve more reliable oxygen supply for general use.	Equipment: one large oxygen concentrator (Linde) with oxygen reservoir, fed into a piped oxygen supply system. Maintenance: provided by local agents of manufacturer. Clinical training: not described.	A retrospective evaluation reported on equipment function and oxygen access.^22^
1993	Nepal	Setting: 1 government referral hospital and 1 private hospital in Kathmandu. Challenges: limited oxygen cylinder supply (logistic and cost barriers). Aims: to achieve more reliable and affordable oxygen supply for anaesthetic use.	Equipment: two oxygen concentrators (DeVilbiss). Clinical training: not described.	A retrospective evaluation reported on equipment function and use and on clinical outcomes.^23^
1993	Nigeria	Setting: single neonatal ward in referral hospital. Challenges: limited oxygen cylinder supply (logistic and cost barriers); high costs to patients, resulting in prioritization and limited use of oxygen. Assets: skilled staff. Aims: to achieve more reliable and affordable oxygen supply for neonatal use.	Equipment: one oxygen concentrator (Puritan-Bennett); car battery for back-up power. Maintenance: informal, supervised by project lead. Clinical training: informal (details unspecified), including use of pulse oximetry and delivery devices to give oxygen to preterm neonates. Financial: small-scale oxygen insurance scheme set up to distribute cost burden, with small contribution from all patients.	A retrospective evaluation reported on concentrator use, function and cost.^24^
1997	Nepal	Setting: single small, remote Himalayan hospital. Challenges: limited oxygen cylinder supply (logistic and cost barriers). Aims: to achieve more reliable and affordable oxygen supply for general use.	Equipment: two oxygen concentrators (CAIRE; DeVilbiss) for general medical use. Clinical training: not described.	A retrospective evaluation reported on equipment use, function and cost^25^
1998	Gambia	Setting: single rural mission hospital. Challenges: severe limitations in electricity (available for only a few hours at night using generator); limited oxygen cylinder supply (logistic and cost barriers). Assets: prior experience with solar power; established equipment maintenance system. Aims: to provide reliable power to enable uninterrupted use of oxygen concentrator.	Equipment: one oxygen concentrator (unspecified); hybrid solar power supply (24 × 90 W PV panels; 6 × 150 Ah 12 V batteries). Maintenance: 1-day maintenance training for staff (details unspecified), plus detailed maintenance schedule. Clinical training: not described. Supervision: visits by project team every 3-6 months.	A retrospective evaluation reported on equipment function and cost implications.^26^
1999	Pakistan	Setting: single military hospital in remote mountain area. Challenges: limited oxygen cylinder supply (logistic and cost barriers). Assets: reliable hydroelectric power; well-trained and equipped staff. Aims: to achieve more reliable and affordable oxygen supply for anaesthetic use and reduce use of cylinders.	Equipment: one oxygen concentrator (DeVilbiss). Clinical training: not described.	A before-and-after study reported on oxygen consumption and clinical outcomes.^27^
1999	Senegal	Setting: single rural hospital. Challenges: limited oxygen cylinder supply (logistic and cost barriers). Aims: to achieve more reliable and affordable oxygen supply for general use.	Equipment: two oxygen concentrators (DeVilbiss; 1 unspecified); oxygen cylinder for back-up. Clinical training: basic training of nurses and technicians (details unspecified).	A project evaluation reported on equipment use, function and costs and on project outcomes.^28^
2000	Gambia	Setting: single referral hospital. Challenges: limited oxygen cylinder supply (logistic and cost barriers); limited technician capacity and maintenance structures. Aims: to improve oxygen supply for general use.	Equipment: > 20 refurbished oxygen concentrators (unspecified), donated (donor not stated). Clinical training: not described.	A qualitative and technical evaluation provided data on equipment use and function and reported on issues.^29^
2013	Sierra Leone	Setting: single district hospital paediatric ward. Challenges: poor power supply (outages for weeks in succession) was main barrier to providing oxygen therapy from existing concentrators. Assets: all staff had received WHO emergency triage and treatment training in the previous year. Aims: to provide reliable power to enable uninterrupted use of oxygen concentrator.	Equipment: two oxygen concentrators (unspecified); solar power supply (18 × 200 W PV panels; 16 × 225 Ah 12 V batteries). Clinical training: basic oxygen training for clinical staff (details unspecified) along with simple wall-charts.	A before-and-after study reported on clinical outcomes.^30^
2013	Uganda	Setting: single paediatric intensive care unit in referral hospital. Challenges: limited existing hydroelectric power supply (unavailable 10% of the time). Assets: access to pulse oximeters; skilled staff. Aims: to provide reliable power to enable uninterrupted use of oxygen concentrator.	Equipment: one oxygen concentrator (unspecified); 3 kVA solar power supply (25 × 80 W PV panels at 48 V; 8 × 220 Ah 12 V batteries); backup oxygen cylinder. Clinical training: not described. Guidelines: a clinical oxygen therapy protocol was introduced.	A prospective evaluation reported on equipment and clinical outcomes.^31^^,^^32^

PV: photovoltaic; WHO: World Health Organization.

Notes: Small-scale projects were those exploring the utility of improved power supplies for oxygen concentrators, including solar power and battery storage. Equipment manufacturers: Airsep, Buffalo, United States of America (USA) (subsidiary of Chart Inc.); CAIRE, San Diego, USA (subsidiary of Chart Inc.); DeVilbiss Healthcare, Mannheim, Germany (subsidiary of Medical Depot Inc.); Healthdyne, Marietta, USA; Longfei, Yueqing, China; Linde Aktiengesellschaft, Munich, Germany; Mountain Medical Equipment Inc. (out of business); Nellcor Puritan Bennett, Boulder, USA (subsidiary of Medtronic plc.); Rimer-Alco, Cardiff, Wales (subsidiary of Air Products and Chemicals Inc.); Simonsen & Weel, Albertslund, Denmark.

**Table 2:** Large-scale interventions to improve oxygen therapy systems in low-resource settings, identified by a systematic literature search

Year	Country	Context	Intervention(s)	Data source(s)
1986	Malawi	Setting: all government hospitals nationally. Challenges: limited oxygen cylinder supply (logistic, cost and supplier barriers); variation in anaesthetic equipment; minimally trained anaesthetic technicians; weak maintenance systems; lack of spare parts and tools. Aims: to achieve uniform oxygen-anaesthetic practice nationally.	Equipment: 104 oxygen concentrators (DeVilbiss) and 44 specially-designed anaesthetic machines (S&W). Maintenance: three maintenance centres; spare parts; tools. Two technicians were trained in Denmark for 3 months; six were trained locally by project team. Supervision: 6 × monthly field visits by technicians from project centre, with retraining. Clinical training: not described. Partners: Malawi government and DANIDA.	Two retrospective technical studies reported equipment use and function, and cost data.^33^^,^^34^
1994	Egypt	Setting: 13 district hospitals in upper Egypt. Challenges: limited oxygen cylinder supply (logistic and cost barriers). Aims: to improve oxygen access and thereby reduce childhood pneumonia deaths.	Equipment: 22 oxygen concentrators (DeVilbiss; Healthdyne; Puritan-Bennett). Maintenance: multidisciplinary team approach. Basic equipment training was given to a nominated doctor in each facility, who supervised use and care of equipment and completed a logbook. Training was by the project team, including an anaesthetist. Different, detailed training was given to engineers at project centre who visited every 3–4 months (re-training after 1 year). Clinical training: not described. Supervision: central coordination team conducted regular field visits. Partners: Egypt health ministry’s Child Survival Project with WHO and USAID.	A prospective evaluation reported on concentrator use and function at 12 months, and on general user feedback.^35^
1990s	Mongolia	Setting: all government hospitals nationally. Aims: not stated	Equipment: 108 oxygen concentrators (DeVilbiss; Healthdyne) procured in the mid-1990s and an additional 100 concentrators in 2001 (hospitals also sourced concentrators independently; DeVilbiss; Healthdyne; Longfei; Airsep; unspecified). No uniformity in procurement or maintenance structures. Clinical training: not described. Guidelines: translation and distribution of WHO oxygen guidelines. Partners: Mongolian health ministry, local NGO and UNICEF.	A cross-sectional survey in 2007 evaluated equipment function in nine district and subdistrict hospitals.^36^
2000	Malawi	Setting: 25 district and regional government hospitals. Challenges: low baseline clinical knowledge and skills of staff; poor access to oxygen; lack of guidelines; poor clinical documentation. Assets: initial sites were chosen if they had a functioning programme for management of acute respiratory infection or integrated management of childhood illness; and commitment from the district health office. Aims: to improve pneumonia case management and thereby reduce childhood pneumonia deaths. This was based on the theory that improved clinician capacity (skill and knowledge), together with a system that enables clinicians to follow guidelines (medications, equipment, support), would lead to improved quality of care (as per guidelines) and thus better clinical outcomes.	Strategy: based on the International Union Against Tuberculosis and Lung Disease (The Union) health-service delivery model, including political commitment; standardized diagnosis and treatment; clinical training; improved medication access; and recording and reporting child pneumonia outcomes. Improved oxygen systems were added to the initial plan, starting in 2002. Guidelines: an inpatient recording form for every child with pneumonia was introduced as both a clinical algorithm and a data collection form. Equipment: 26 oxygen concentrators (DeVilbiss), with flow-splitters in paediatric wards and 2–3 years’ supply of spare parts. Clinical training: comprehensive pneumonia-care training, including oxygen-related training, for nurses and medical officers (5-day course). Training was by project team at central location. Coverage was high (about 25% of health workforce), but follow-up in-service training failed. Maintenance: trained technicians from health ministry. Intention was to provide 3-monthly maintenance visits, but few visits were conducted, activities were not documented, and spare parts were lost.^37^ Partners: Malawi health ministry, The Union, the Bill & Melinda Gates Foundation and the Government of Scotland.	A nonrandomized field trial^38^ reported clinical outcomes. Long-term clinical and quality of care data were obtained from retrospective audits^39^^,^^40^ and qualitative studies,^41^^–^^44^ and provided by various reports^45^^,^^46^ and a thesis.^47^ An external evaluation^37^ and a cross-sectional assessment of concentrators^36^ provided equipment data.
2004	Gambia	Setting: four hospitals and health centres. Challenges: limited oxygen access. Assets: well-trained biomedical engineers. Aims: to achieve more reliable and affordable oxygen supply for paediatric use.	Equipment: 27 oxygen concentrators (Airsep) introduced over 7 years, with uninterruptable power supply. Pulse oximetry was introduced later. Maintenance: computer-based record system; 3-monthly visits by trained biomedical engineers; preventive maintenance protocol for users. The maintenance structure was effective, with maintenance visits occurring 3–4 times annually (even to the most distant facilities) and accurate logbook completion for 8 years of follow-up. Clinical training: not described. Partners: the Medical Research Council of the Gambia	An audit^48^ and implementation report^49^ provided data on equipment use and function, cost and user feedback. Data on context came from needs analyses.^50^^,^^51^
2005	Papua New Guinea	Setting: five district and provincial hospitals in the highlands and coastal areas of Papua New Guinea. Challenges: limited oxygen access. Pneumonia was the leading cause of child mortality in the country, and the burden of disease was particularly high in the remote, high-altitude, highland villages. Assets: adequate power supply; motivated senior staff. Aims: to improve quality of pneumonia care and thereby reduce childhood pneumonia deaths. This was based on principles of quality improvement and participatory development with minimal external support.	Equipment: 15 oxygen concentrators (Airsep) in paediatric wards, with flow-splitters and pulse oximeters (and careful procurement of equipment, tools and adequate spare parts). Pulse oximetry (with user-training) was introduced 1 year before intervention, to build skills and awareness about hypoxaemia and oxygen therapy, and to obtain accurate baseline data. Clinical training: for nurses and medical officers, on-site by local paediatricians, focusing on pneumonia and the use of oxygen. Guidelines: clinical guidelines and maintenance protocols. Supervision: multidisciplinary team provided oversight at national level, with regular support visits to participating hospitals; participatory data collection, analysis and action involving hospitals and health ministry. Other: emphasis on patient-centred care; attention to the work environment (e.g. high-dependency areas for grouping the sickest children together). Partners: Papua New Guinea National Department of Health and WHO.	A before-and-after effectiveness study^52^ reported clinical outcomes and cost-effectiveness. Project evaluations^53^^–^^55^, and hypoxaemia studies;^3^^,^^56^ provided baseline epidemiological and contextual data. Two modelling papers reported cost data.^57^^,^^58^
2011	Lao People's Democratic Republic	Setting: 10 district hospitals across five provinces. Challenges: hospitals operated on a user-pay system, and cost of oxygen was seen as the major barrier to patient access. Assets: adequate power supply; support from hospital leadership (10 control hospitals were from the same province). Aims: to provide a more cost-effective oxygen solution to hospitals, enabling hospitals to reduce the cost burden on patients; to enable clinicians to provide oxygen to all children and neonates who need it; and to achieve better clinical outcomes.	Equipment: 3–6 oxygen concentrators (Airsep) per hospital with pulse oximeters and a Sureflow flowmeter assembly (Airsep), enabling individual titration of oxygen from a single concentrator to up to five patients simultaneously; nasal prongs; and an oxygen analyser to test the concentrator oxygen purity. Maintenance: training for central and provincial engineers and a technician from each hospital was provided by biomedical engineer at a central location. Clinical training: 2-day practice-based training at each hospital by project team, including a paediatrician. Training material was translated into Lao. Financial: through a collaborative process, all hospitals decided to make oxygen from concentrators freely available to all patients. Supervision: supervision visits by coordinators (at 3, 12 and 24 months). Partners: Lao People's Democratic Republic health ministry, WHO and Japanese government.	A controlled before-and-after study reported on: clinical outcomes; quality of care; and implementation data (Gray AZ et al., CICH, unpublished data, 26 January 2017; Morpeth M et al., CICH, unpublished data, 26 January 2017). Project reports provided additional contextual and implementation data.^59^^–^^61^

CICH: Centre for International Child Health (Australia); DANIDA: Danish International Development Agency; NGO: nongovernmental organization; UNICEF: United Nations Children’s Fund; USAID: United States Agency for International Development; WHO: World Health Organization.

Notes: Large-scale projects involved complex interventions targeting technicians, clinicians and often administrators and policy-makers. Equipment manufacturers: Airsep, Buffalo, United States of America (USA) (subsidiary of Chart Inc.); DeVilbiss Healthcare, Mannheim, Germany (subsidiary of Medical Depot Inc.); Healthdyne, Marietta, USA; Longfei, Yueqing, China; Nellcor Puritan Bennett, Boulder, USA (subsidiary of Medtronic plc.); Simonsen & Weel, Albertslund, Denmark.

**Table 3:** Impact of improved oxygen therapy systems on inpatient child mortality from studies identified in a systematic literature search

Country, year	Total sample size No.	Before intervention			After intervention		Absolute risk reduction, %	Unadjusted RR or OR (95% CI)	EPHPP study quality rating^16^
		Children treated No.	No. (%) of deaths		Children treated No.	No. (%) of deaths			
**Lao People's Democratic Republic, 2011**^a^									
Pneumonia deaths									
Control group	1 355	712	12 (1.7)		643	14 (2.2)	−0.5	1.29 (0.60–2.77)^b^	Moderate
Intervention group	1 412	711	19 (2.7)		701	6 (0.9)	1.8	0.32 (0.13–0.80)^b^	Moderate
**Malawi, 2000**^38^									
Pneumonia deaths	47 228	389	73 (18.8)^c^		47 228	4 605 (9.8)^d^	NA^c^	0.79 (0.64–0.99)^e^	Weak
**Papua New Guinea, 2005**									
Pneumonia deaths^52^	11 291	7 161	356 (5.0)		4130	133 (3.2)	1.8	0.65 (0.52–0.78)^b^	Moderate
Other deaths^54^	21 044	13 354	778 (5.8)		7690	348 (4.5)	1.3	0.79 (0.70–0.89)^b^	Weak
**Sierra Leone, 2013**^30^									
All-cause deaths	1 822	920	34 (3.7)		902	16 (1.8)	1.9	0.48 (0.27–0.86)^b^	Weak

CI: confidence interval; EPHPP: Effective Public Health Practice Project; NA: not available; OR: odds ratio; RR: relative risk.

^a^ Morpeth M et al., CICH, unpublished data, 26 January 2017.

^b^Relative risk.

^c^ Data from year 2000. Malawi case-fatality rate in 2000 was derived from only 3 months of data; the rate for all of 2001 was 13.3%.

^d^ Data from year 2005.

^e^ Odds ratio. Malawi investigators used stepped-wedge methodology. The chronological data did not represent actual before-and-after intervention phases; the odds ratio was derived using unadjusted statistical methods.

Notes: Studies were restricted to children aged < 5 years admitted to the paediatric ward. All studies used a before-and-after intervention study design.

Overall, projects used a variety of strategies to improve oxygen therapy. We identified five themes (Box 2), each containing multiple mechanisms that explain how interventions to improve oxygen therapy work (or not) in different contexts. We tested these theories using data from all included projects and identified key factors reducing or enhancing the efficacy of oxygen therapy projects (Box 3).

Box 2 **Theoretical themes identified to explain how interventions to improve oxygen therapy systems in low-resource settings might work**

Theme 1 (oxygen access) recognizes that lack of oxygen in health-care facilities is a major barrier to care, and proposes that making oxygen therapy available will enable more children to be treated and with better outcomes. These projects typically emphasized the importance of quality oxygen equipment and effective maintenance programmes and some also addressed financial barriers to access.

Theme 2 (oxygen use) recognizes that health-care workers may not use oxygen effectively, and proposes that building the capacity and motivation of health workers will enable children to be treated appropriately and have better outcomes. These projects typically emphasized training and supervision of clinical staff, including retraining and follow-up to ensure sustainability.

Theme 3 (broader care practices) recognizes that broader issues of quality of care impact on clinical outcomes, and proposes that strengthening these processes will enable higher quality of care and better outcomes. These projects emphasized broader quality of care issues, such as supply of essential medications, clinical review and feedback and record-keeping.

Theme 4 (supportive management) recognizes that managerial and political support is essential, and proposes that engagement with managers and policy-makers will support successful implementation and sustainability. These projects emphasized the engagement and responsibility of stakeholders at district, regional and national level in planning, implementation and evaluation.

Theme 5 (hospital team) recognizes that hospital-level responsibility and action influences success, and proposes that enhancing the motivation of the hospital team will drive improvements in the use of oxygen and the care of equipment. This theme has not been overtly reported in previous oxygen therapy projects, but was frequently cited in interviews as a strong predictor of success by those involved.

Note: Potential theoretical themes were identified in the first stage of the realist review by a preliminary scan of the literature and interviews with key experts and stakeholders involved in oxygen projects. Themes were reviewed and refined during the extraction and analysis of data.

Box 3 **Contextual and programmatic factors that influenced the efficacy of interventions to improve oxygen therapy systems in low-resource settings, positively (+) or negatively (−)**

**Contextual factors**

Strong evidence across multiple studies:

(+) Target hospital has unmet need for oxygen (e.g. high prevalence of pneumonia or hypoxaemia);

(+) Target hospital has limited access to oxygen (though not necessarily no access);

(+) Hospital managers recognize the need for improved oxygen systems;

(+) Functional maintenance system in place, with ability to meet additional needs of oxygen equipment (particularly regular preventive maintenance);

(+) Ward environment and culture facilitates good oxygen therapy practices (e.g. adequate space, patient-centred care, good interdisciplinary relationships);

(+) Hospital power supply is adequate;

(−) High turnover of skilled clinicians (without transfer of knowledge);

(−) Weak existing maintenance system is unable to meet additional needs of oxygen equipment.

Limited evidence from one or few studies:

(+) Target hospital starts from low baseline of clinical care (i.e. has more possibility to show improvement);

(+) Hospital staff members have some experience using oxygen;

(−) Oxygen is expensive to patients.

**Programmatic factors**

Strong evidence across multiple studies:

(+) Clinician in ward leadership role supervises and supports oxygen use;

(+) Uniform procurement of oxygen equipment that is high quality and appropriate for the environmental conditions;

(+) Skilled technicians, and spare parts and tools, are available for installation and maintenance of equipment;

(+) Ongoing cost of oxygen to hospital is adequately financed;

(+) Hospital is the recipient of financial benefits (from improved oxygen system) and has control over future spending on oxygen therapy;

(+) Training is practical and adapted to the hospital’s specific needs (preferably conducted on-site);

(+) Clinical use of oxygen and pulse oximetry is monitored and deficiencies are addressed;

(+) Problems related to unreliable power supply are resolved;

(−) Lack of skilled technicians and tools and spare parts;

(−) Weak support of maintenance team by responsible body.^a^

Limited evidence from one or few studies:

(+) Hospital leaders are involved in needs assessment, planning, implementation and review;

(+) Quality, user-friendly equipment is available to detect hypoxaemia (pulse oximetry);

(+) Oxygen analyser available to assess oxygen concentrator function;

(+) Quality, user-friendly equipment delivery devices are available (including safe mechanism for titrating oxygen flow to individual patients);

(+) Well organized hospital multidisciplinary team is responsible for oxygen therapy activities;

(+) Hospitals are supported to measure outcomes of therapy and respond (i.e. quality improvement);

(+) Broader aspects of quality of care are improved (e.g. medical supplies, record-keeping);

(+) Costs of oxygen therapy to patients are minimized;

(+) Country’s health ministry (or similar) provides ongoing support for activities related to oxygen therapy;

(−) Training of clinicians is not done on-site;

(−) Maintenance capacity is not made available locally.

Possible factors that might be tested in future studies:

(+) Capacity of health workers to pass on knowledge and skills is developed.

^a^ Responsible body could be the hospital management, or state or federal government department, or a nongovernmental organization.Notes: The factors were identified from a systematic literature search and analysis of 45 studies from 20 projects in 15 countries, involving intervention(s) to improve oxygen therapy systems (Table 1 and Table 2). All projects involved the introduction of oxygen therapy equipment. Many projects also included additional interventions targeting technicians, clinicians, administrators and policy-makers. Contextual refers to factors in the environment in which the project was introduced that influence the success of the project, such as characteristics of participating health facilities and existing capacity for oxygen therapy. Programmatic refers to factors about the intervention or programme itself that influence success, such as what was done and how it was implemented.

### Oxygen access

Increasing the availability of oxygen therapy to patients was an implicit aim in virtually all the intervention projects we studied, but few studies evaluated it. Hospitals in most projects had some access to oxygen cylinders (Gray et al.),^21^^,^^22^^,^^24^^–^^26^^,^^35^ or concentrators^50^ before the intervention, but supply was limited by depletion of cylinders (which are costly and difficult to refill) and broken equipment. The only baseline data on oxygen availability to patients were from Papua New Guinea, where oxygen was available for 326 (87%) of 375 children who had hypoxaemia on admission.^56^

Two large-scale projects reported increased access to oxygen cylinders or concentrators compared with the baseline.^46^^,^^48^^,^^50^ Most projects reported increased oxygen use (more patients given oxygen or greater volume of oxygen provided to patients; Morpeth et al.),^19^^,^^24^^,^^25^^,^^28^^,^^34^^–^^37^^,^^48^^,^^49^^,^^53^ while some reported fewer referrals for hypoxaemia (Gray et al.),^26^^,^^34^ and decreased dependence on oxygen cylinders (Gray et al.).^21^^,^^22^^,^^24^^–^^27^^,^^34^^–^^36^^,^^49^ Data from Papua New Guinea suggested that hospitals with poorer access to oxygen (and pulse oximetry) at baseline achieved greater reductions in pneumonia case-fatality rates post-intervention than hospitals with reasonable access at baseline.^52^^,^^56^

We identified three major determinants of oxygen availability: (i) equipment; (ii) maintenance; and (iii) affordability.

#### Equipment

Although the provision of quality, user-friendly equipment is necessary for an improved oxygen therapy system, it is not easy to achieve. Baseline data from several projects documented poor quality and broken equipment that had often been donated by international donors but did not suit the needs of its users or the environmental conditions.^29^^,^^48^^–^^50^^,^^53^^,^^59^ Studies consistently reported the importance of procuring oxygen concentrators that were proven to work in hot and humid conditions. This was based on observations of maintenance problems and premature equipment failure when projects provided untested concentrators or used a variety of concentrator types (Gray et al.).^28^^,^^36^^,^^46^^,^^51^^,^^53^

Pulse oximeters were not used in most of the oxygen projects before 2005. Where pulse oximeters were used, they were valued as both a diagnostic aid (determining which children require oxygen therapy) and therapeutic aid (giving staff and patients confidence in oxygen therapy; Gray et al.).^3^^,^^49^^,^^52^^,^^56^ Limited data suggested that the introduction of pulse oximetry can improve oxygen use and reduce pneumonia case fatality rates, even with suboptimal oxygen access.^3^^,^^56^

Oxygen delivery devices varied between projects. Nasal prongs were easiest for health-care workers, and some projects reported confusion when multiple options were provided to inexperienced users.^24^^,^^35^^,^^37^^,^^46^ Some projects used flow-splitters to share oxygen between multiple patients (users would change the size of outlet nozzles to control flow rates), but these were frequently malfunctioning (e.g. missing plugs, blocked tubing) or used incorrectly, resulting in no gas flow to patients.^37^^,^^48^ This led to the development of flowmeter assemblies, allowing individual titration of gas to multiple patients without changing plugs or connections (Gray et al.).^53^^,^^55^

Power supplies were a major factor limiting the use of oxygen concentrators.^49^^,^^51^^,^^55^^,^^57^ Uninterruptable power supply systems are useful if power outages are infrequent (e.g. less than daily) and brief (e.g. less than 20 minutes), but not if power failures are frequent or prolonged.^49^ Solar power with battery storage is feasible, but requires experienced technicians and quality products.^26^^,^^30^^,^^31^

#### Maintenance

For users and technicians to provide reliable equipment maintenance and repair they need the capacity (knowledge and skills); opportunity (transport and access to spare parts); and motivation (role recognition and anticipated benefit). The Gambia provided a positive case study involving: well-trained biomedical engineers; a schedule for preventive maintenance; spare parts; electronic work orders (facilitating access to engineers); and a maintenance protocol for users.^48^^,^^49^ They reported excellent long-term equipment function: 21/27 (78%) of oxygen concentrators working at 8-year follow-up; and minimal equipment down-time (5.2% of total cumulative hours in service).^48^ They identified problems early (e.g. 37/53 (70%) repair needs identified and fixed during preventive maintenance visits) and showed that, while one third of concentrators needed repair each year, most repairs were cheap and simple (annual spare parts cost around 15 United States dollars per concentrator and required 50 person-hours).^48^ Other projects with reasonable maintenance systems also reported good equipment function up to 3 years post-implementation (Gray et al.).^22^^,^^34^^,^^35^^,^^52^^,^^53^ Projects where maintenance systems failed reported high equipment down-time and early equipment malfunction.^21^^,^^29^^,^^36^

Many projects reported deficiencies in maintenance activities (e.g. site visits, spare parts, record-keeping and care by users), resulting in missed opportunities for early repair (Gray et al.).^35^^,^^37^^,^^46^^,^^48^^,^^49^^,^^53^^,^^60^ Projects that integrated maintenance activities into existing (usually government) biomedical maintenance systems achieved success proportional to the quality of the existing system and its capacity to incorporate additional needs (Gray et al.).^34^^,^^35^^,^^46^^,^^53^^,^^60^

#### Affordability

Oxygen must be affordable for patients and hospitals. In user-pay hospitals oxygen was reported to be expensive for patients, contributing to delayed presentation, early discharge, and high patient debt burden (Gray et al.).^24^^,^^60^ In the Lao People's Democratic Republic, oxygen from concentrators was provided free of charge, thus reducing the cost of oxygen to patients and decreasing early discharge rates for unwell patients (Gray et al., Morpeth et al.).^46^ In Nigeria, a neonatal unit used an oxygen insurance scheme to distribute the cost burden among all admitted patients and allow sustainable cost recovery.^24^ Many projects reported that concentrator-based oxygen systems were relatively more cost–efficient than cylinder-based systems.^19^^,^^21^^,^^24^^,^^25^^,^^27^^,^^28^^,^^34^^,^^49^^,^^52^^,^^57^^,^^58^^,^^61^ Demonstrated cost–efficiency can support the sustainability and future expansion of oxygen therapy,^21^^,^^24^^,^^26^^,^^49^ but this may be limited if the decision-making body does not identify both financial and clinical benefits (Gray et al.).^34^^,^^57^^,^^58^

### Oxygen use

All the large-scale oxygen projects, and many single-site projects, aimed to improve how health-care workers used oxygen. However, while the overall use of oxygen increased, it was usually lower than expected based on admission and case-mix data, suggesting underuse of oxygen (Gray et al.).^35^^,^^37^^,^^40^ The highest mean concentrator use (around 15–18 hours/day) was reported by two hospitals with high caseloads and close supervision by project doctors;^24^^,^^28^ other projects reported a mean use of less than 6 hours per day (Morpeth et al.).^19^^,^^25^^,^^34^^,^^36^^,^^37^^,^^48^^,^^49^^,^^53^

Post-implementation data from the Lao People's Democratic Republic, Malawi and Papua New Guinea showed persisting deficiencies in practice (< 50% of children with pneumonia received pulse oximetry; 22–80% of hypoxaemic children received oxygen) with great variation between hospitals (Morpeth et al.).^40^^,^^54^ Hospitals with good pulse oximetry and oxygen practices achieved better clinical outcomes, with greater improvement if they started from a low baseline (Morpeth et al.).^53^

Appropriate oxygen use was influenced by health-workers’: (i) knowledge and skills; (ii) motivation; and (iii) work environment.^62^

#### Knowledge and skills

Low knowledge and skills among health workers was almost universally reported, and many health workers had misconceptions and fears about oxygen therapy that affected their motivation (e.g. fear of the concentrator technology or belief that oxygen therapy caused death). Many projects provided initial training, and these generally showed improved knowledge and skills (Gray et al.).^38^^,^^45^^,^^52^^,^^53^^,^^61^ High training coverage rates and in-service re-training were regarded as important in hospitals that faced chronic staff shortages and high staff turnover.^45^^,^^53^ However, the proportion of staff trained correlated poorly with practice change or clinical outcomes, suggesting that training the right people in the right way may be more important than training more people.^38^^,^^52^^,^^61^

#### Motivation

The greatest reported motivator for staff was witnessing the benefits of oxygen first-hand (Gray et al.).^25^^,^^27^^,^^28^^,^^35^^,^^37^^,^^46^ Challenges to motivation included: confusion about equipment and guidelines; associating oxygen therapy with death; technical difficulties; and competing workplace priorities (Gray et al.).^25^^,^^27^^,^^28^^,^^35^^,^^37^^,^^41^^–^^44^^,^^46^ This was best addressed by practical on-site training addressing specific challenges and needs (Gray et al.);^53^ and regular on-site supervision (Gray et al.).^24^^,^^25^^,^^27^^,^^28^^,^^35^^,^^37^^,^^46^^,^^48^^,^^53^

#### Work environment

The physical and social environment could enable or inhibit good practice in oxygen therapy. For example, creation of high-dependency spaces within wards prioritized care for sick patients; endorsing protocols created new practice norms; wall-charts reminded users about oxygen use; logbooks facilitated regular equipment maintenance; flowmeter assemblies enabled oxygen delivery to multiple patients; pulse oximetry enabled identification of hypoxaemic children, helped involve families in care and overcame fears about oxygen; and oxygen analysers gave users confidence that the concentrator worked (Gray et al.).^28^^,^^35^^,^^46^^,^^48^^,^^49^^,^^53^

### Broader care practices

The projects in the Lao People's Democratic Republic, Malawi and Papua New Guinea embedded improved oxygen systems within broader measures to improve quality of care. All reported changes that extended beyond oxygen-related care, such as improved staffing; reaching neglected populations; strengthening supply chains; and addressing other care deficits (Gray et al.).^46^^,^^53^ While it was not possible to separate the effects of these quality of care interventions from the effects of the oxygen-related interventions, hospitals that improved the broader aspects of care typically reported the biggest improvements in outcomes (Gray et al.).^46^^,^^52^^,^^53^^,^^56^

### Supportive management

All the large-scale oxygen therapy projects required some degree of regional and national government support. In Egypt, the Lao People's Democratic Republic and Papua New Guinea effective relationships with government biomedical departments and the support of a multidisciplinary national oxygen team strongly facilitated equipment maintenance (Gray et al.).^35^^,^^53^ In Malawi, however, maintenance failed due to lack of support and supervision of the biomedical team.^46^ Support from people within the Malawian health ministry enabled the programme to be sustainably incorporated into government child health programmes.^35.32 ^However, sustainability failed in Mongolia, partly due to the loss of key health ministry staff. In the Lao People's Democratic Republic and Papua New Guinea, ongoing training has been supported by professional organizations and some regional health authorities, but sustainable programme implementation and scale-up has been limited by financial and practical constraints at the policy level (Gray et al.).^53^

### Hospital team

Investigators from many projects observed that ownership and acceptance of the project by hospital staff was a key determinant of success, enhancing: responsive problem-solving; equipment care and maintenance; clinical use of oxygen; continuity of knowledge and skills; and general quality of care (Gray et al.).^35^^,^^46^^,^^48^^,^^53^ Limited data suggests that hospital-level ownership was greater when staff were convinced of the potential benefits of improved oxygen systems, and were actively involved in oxygen improvement activities as a multidisciplinary team.^24^^,^^28^^,^^52^

Achieving hospital-level ownership may be more challenging for large-scale programmes that are initiated externally. A common approach was to find a responsible person at each hospital who would be the primary contact and local project leader. When it worked, this strategy created a local champion who had ready access to technical expertise and local staff, and ideally shared responsibility with a multidisciplinary team (Gray et al.).^28^^,^^46^^,^^48^^,^^53^ This failed if the responsible person left their employment or lacked the power to effect change (Gray et al.).^46^

While external agencies often brought funding and technical expertise to oxygen projects, studies reported that agencies could also be disruptive influences on hospital and health systems (Gray et al.).^29^^,^^46^^,^^52^ Success depended on effective local participation (in planning, implementation and evaluation); clear communication between stakeholders (including priorities, roles and responsibilities); and ongoing support of local implementers (Gray et al.).^29^^,^^46^^,^^52^

## Discussion

The safe and effective provision of oxygen is a challenge for doctors, hospital administrators and government officials globally. Typically, some oxygen therapy equipment is available, but without the maintenance capacity to keep it functioning or the clinical expertise to use it effectively (Gray et al.).^36^^,^^40^^,^^46^^,^^50^^,^^56^^,^^63^ While WHO guidelines offer advice on the technical specifications^9^ and the clinical application of oxygen,^8^ there has been little guidance on how to bring about changes in practice. In shifting the focus from what works to what happens,^64^ realist review methods hold great promise for improving our understanding of how to bridge the implementation gap.^65^ This is relevant for oxygen, and many other similarly simple, life-saving therapies that countries are struggling to scale up.

**Fig. 2:**
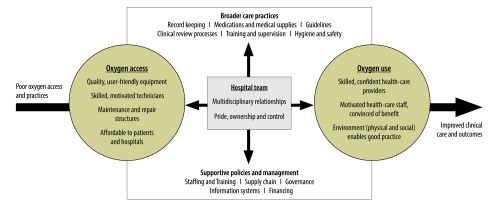
Improving outcomes with effective oxygen therapy: a framework depicting the key requirements of an effective oxygen system

We identified a multilayered array of mechanisms that need to work together to achieve results. In broad terms, hospitals need good access to oxygen and effective use of oxygen, which should be facilitated by a broad quality improvement capacity, a strong managerial and policy support and multidisciplinary teamwork (Fig. 2). Within each domain, additional mechanisms are at play. It is the interaction of these processes, together with various contextual factors that determines the outcomes.

O_2_: oxygen.

Note: The framework was devised from a systematic literature search and analysis of 45 published studies from 20 projects in 15 countries, involving intervention(s) to improve oxygen therapy systems (Table 1 and Table 2).

This framework is work in progress and it needs further testing. While there is substantial evidence to support some aspects of this framework, there is little evidence to support other aspects. Important future questions include: (i) the role of various policy-makers and managers in supporting the effective use of oxygen; (ii) the function of a multidisciplinary hospital oxygen team; (iii) the influence of broader care practices and potential role of quality improvement teams; (iv) the role of alternative energy sources in ensuring adequate power; and (v) how to integrate pulse oximetry and good oxygen practices more effectively into routine care. Those who are implementing an oxygen therapy system should not only evaluate the effectiveness of their programme, but test the mechanisms through which their programme works.

Our review had some limitations. First, realist reviews cannot explore all potential theories or all contextual influences.^11^ We decided to include a wide range of interventions, but prioritized the exploration of theories that were most generalizable and applicable to low-resource hospitals providing general care for children. Our search criteria were therefore broad and inclusive. However, we may not have identified all unpublished studies. Second, realist reviews accept a broad array of data but this cannot be exhaustive.^11^ We used published and unpublished evaluation reports, and interviews with study authors to clarify particular theories for which evidence was lacking. Different investigators, from different projects, may have provided different information. However, we do not believe this would have shifted the weight of evidence substantially, and we have documented where evidence is still lacking. Indeed, we believe that the validity of our findings was strengthened by exposing the data to critique from the implementers and other stakeholders of all the large-scale projects identified.

In conclusion, our framework offers a practical, evidence-based approach to improving oxygen therapy in low-resource settings and may be relevant for other programmes involving the introduction of health technologies. It will assist practitioners and policy-makers to evaluate their current system(s), and create solutions that are appropriate for their context. It will assist implementers to understand and optimize their activities, providing both the flexibility and structure to adapt to different settings and respond to evolving needs.
